# Supplementary material for: Proof of concept for real-time detection of SARS CoV-2 infection with an electronic nose
Source: PLoS One. 2021 Jun 2;16(6):e0252121. doi: 10.1371/journal.pone.0252121 (PMC8172018; doi:10.1371/journal.pone.0252121)
Supplement: S1 File — (PDF) [file pone.0252121.s003.pdf]

Supplementary Materials for:

Proof of Concept for Real-Time Detection of SARS CoV-2 Infection with an Electronic Nose

Kobi Snitz[1], Michal Andelman-Gur[1], Liron Pinchover[1], Reut Weissgross[1], Aharon Weissbrod[1], Eva Mishor[1], Roni Zoller[1], Vera Linetsky[1], Abebe Medhanie[1], Sagit Shushan[1,2], Eli Jaffe[3], Noam Sobel[1]

[1]Department of Neurobiology and Azrieli Center for Human Brain Imaging and Research, Weizmann Institute of Science, Rehovot, Israel. [2]Department of Otolaryngology & Head and Neck Surgery, Edith Wolfson Medical Center, Holon, Israel. [3]Magen David Adom in Israel and Department of Emergency Medicine, Ben-Gurion University of the Negev, Beer Sheva, Israel.

In addition to this document, the supplementary materials include an online video and a zipped directory including all the data and code

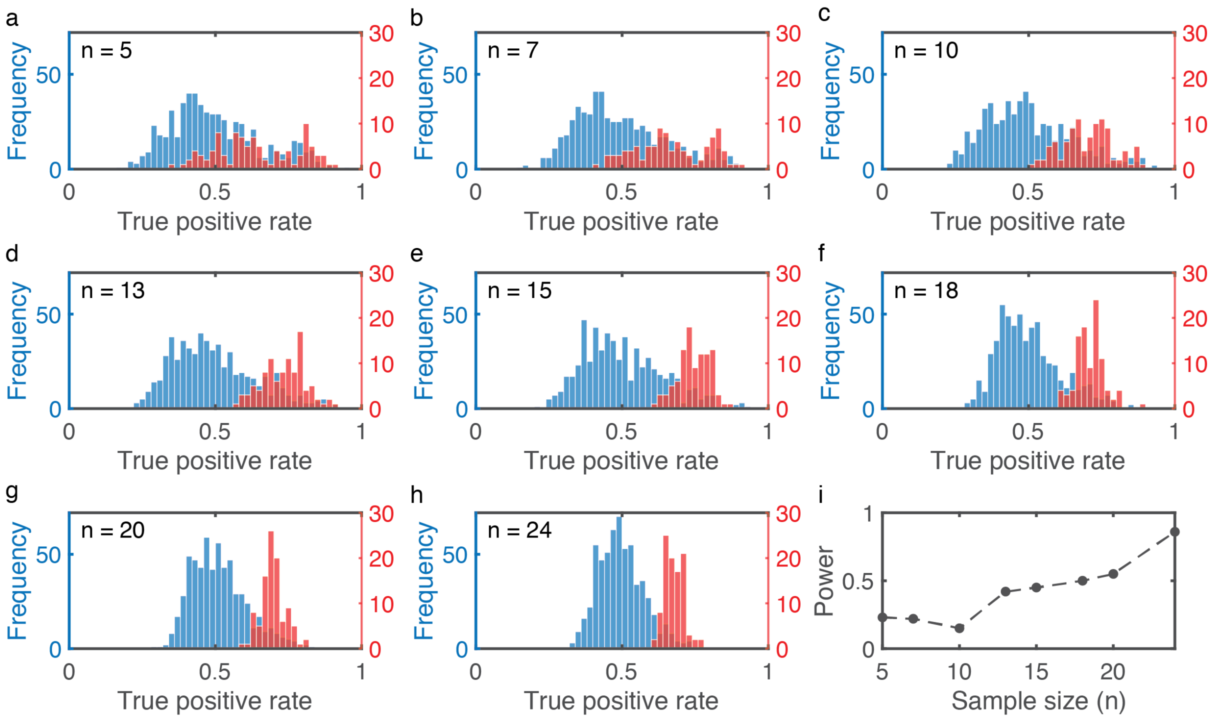

Supplementary Figure 1: Power at different sample sizes

To calculate the power of our experiment with 27 positive participants we performed the 600-fold bootstrap described in the text for assigning a p value, but now using samples of different size. Thus, the power of the experiment at 5 positive samples was the proportion of selections of 5 positive samples which resulted in significant classifier performance. And so on for 7,10,13, and so on.

Supplementary Video Online

A video demonstrating activity at the drive-through. The person being tested is an experimenter demonstrating, and not a participant.

Supplementary Materials Data and Code folder

A folder containing all the raw data, the code, and a readme file explaining how to run it.
