# Supplementary material for: Proof of concept for real-time detection of SARS CoV-2 infection with an electronic nose
Source: PLoS One. 2021 Jun 2;16(6):e0252121. doi: 10.1371/journal.pone.0252121 (PMC8172018; doi:10.1371/journal.pone.0252121)
Supplement: Attachment — Submitted filename: EnoseReview.pdf [file pone.0252121.s004.pdf]

Review of "Proof of Concept for Real-Time Detection of SARS CoV-2 Infection with an Electronic Nose"

Manuscript Number: PONE-D-20-27895

Page 4 lines 8-9 state, "When a compound interacts with the sensor, this results in an oxygen exchange that leads to decreased electrical conductivity." This would appear to be inaccurate, as the sensor's conductivity increases as the target gas concentration increases which may be confirmed by simply referring to the sensor manufacturer's technical data sheet. Also, the usefulness of this paper would be greatly increased by specifically stating the VOCs in question and providing some technical data sheet information on the sensors as supplemental material.

It is currently well known in the art of breath analysis detection that a unique VOC pattern is emitted from healthy individuals as well as individuals having a disease. It is also well known in breath collection protocols that environmental contamination of breath samples is a key problem that is required to be addressed in breath sample collection for maintaining the integrity of the collected sample and for facilitating accuracy of the study results.

The authors teach participant's breath samples were taken in the participant's vehicle. Page 6, Lines 19-21 teach, "The participant was then handed the sampling valve, and instructed to hold it snugly against a nostril opening for 80 seconds. The participants were told to breathe normally through their open mouth during these 80 seconds". The problem with this procedure set forth by the authors is that a normal breathing pattern, in my opinion, is not conducive to accurately detect endogenous VOCs, whereby, a deep breath with a long exhalation will ensure that critical VOCs retained deep within the lungs are expelled during the collection.

Further, a breath sample from a single nostril presents opportunity of contaminants from entering the breath collection vessel from environmental contaminants being inhaled through the second nostril and/or the mouth, which was taught by authors to have been exposed to environmental air which inherently contain contaminants such as including, but not limited to, car exhaust, air fresheners, and/or breath from a second individual sitting in the same vehicle. Both the second nostril and the mouth should have been retained in an enclosed device such as a sealed mask to be completely sealed off from the aforesaid environmental contaminants. This would, in my opinion, been a much better protocol and would not call the results into question.

The reviewer has determined the Authors have completely disregarded standard breath collection protocols. Thus, it is my opinion that the integrity of this study has been compromised, rendering the data presented to be unsatisfactory for its intended purpose.
